# Cutaneous protothecosis in a dog successfully treated with oral itraconazole in pulse dosing

**DOI:** 10.1186/s13028-022-00662-x

**Published:** 2023-02-21

**Authors:** Vanessa Cunningham Gmyterco, Tomasz Jagielski, Gustavo Baldasso, Louise Helene Bacher, Márcio Garcia Ribeiro, Marconi Rodrigues de Farias

**Affiliations:** 1 Department of Veterinary Medicine, School of Life and Sciences, Pontifícia Universidade Católica do Paraná, 1155 Imaculada Conceição Street, Curitiba, PR 80215-901 Brazil; 2 Department of Medical Microbiology, Institute of Microbiology, Faculty of Biology, University of Warsaw, I. Miecznikowa 1, 02-096 Warsaw, Poland; 3 Department of Animal Production and Preventive Veterinary Medicine, School of Veterinary Medicine and Animal Sciences, Sao Paulo State University-UNESP, Botucatu, SP 18618-681 Brazil

**Keywords:** CYTB gene, Genetic identification, MALDI-TOF MS, *Prototheca* sp., *Prototheca wickerhamii*

## Abstract

**Background:**

Protothecosis is a rare infectious disease caused by unicellular, achlorophyllous, microalgae of the genus *Prototheca*, ubiquitously distributed in nature. The algae are emerging pathogens, whose incidence is increasing in both human and animal populations and serious systemic infections related to this pathogen have been increasingly described in humans in recent years. After mastitis in dairy cows, canine protothecosis is the second most prevalent form of the protothecal disease in animals. Here, we report the first case of chronic cutaneous protothecosis due to *P. wickerhamii* in a dog in Brazil, successfully treated with a long-term therapy with itraconazole in pulse.

**Case presentation:**

Upon clinical examination, exudative nasolabial plaque, ulcered, and painful lesions in central and digital pads and lymphadenitis were observed in a 2-year-old mixed-breed dog, with a 4-month history of cutaneous lesions and contact with sewage water. Histopathological examination revealed intense inflammatory reaction, with numerous spherical to oval, encapsulated structures stained with Periodic Acid Schiff, compatible with *Prototheca* morphology. Tissue culture on Sabouraud agar revealed yeast-like, greyish-white colonies after 48 h of incubation. The isolate was subjected to mass spectrometry profiling and PCR-sequencing of the mitochondrial cytochrome b (CYTB) gene marker, leading to identification of the pathogen as *P. wickerhamii*. The dog was initially treated with oral itraconazole at a dosage of 10 mg/kg once daily. After six months, the lesions resolved completely, yet recurred shortly after cessation of therapy. The dog was then treated with terbinafine at a dose of 30 mg/kg, once daily for 3 months, with no success. The resolution of clinical signs, with no recurrence over a 36-months follow-up period, was achieved after 3 months of treatment with itraconazole (20 mg/kg) in pulse intermittently on two consecutive days a week.

**Conclusions:**

This report highlights the refractoriness of skin infections by *Prototheca wickerhamii* with therapies proposed in the literature and suggests a new treatment option with oral itraconazole in pulse dosing for long-term disease control successfully performed in a dog with skin lesions.

## Background

Protothecosis refers to a localized or disseminated disease of humans and animals caused by unicellular, colourless, yeast-like microalgae of the genus *Prototheca*. These microorganisms are normally saprophytic and ubiquitous in nature with a predilection for wet and organic-rich areas [1, 2]. The current *Prototheca* taxonomy, based on the mitochondrially-encoded *CYTB* gene marker, accepts fourteen species of which those exhibiting pathogenic potential are *Prototheca wickerhamii*, *P. bovis* (formerly *P. zopfii* genotype 2), *P. ciferrii* (formerly *P. zopfii* genotype 1), *P. cutis*, *P. miyajii* and *P. blaschkeae* [3]. In humans, protothecosis has often been associated with underlying medical conditions and immunosuppression [4–6]. The most prevalent form of the protothecal disease in animals is bovine mastitis, typically manifested as chronic and drug-resistant infections resulting in significant reductions in milk production and premature culling [7]. After dairy cattle, dogs are the second animal species most usually affected. The infection usually starts following ingestion of contaminated water or feed or at a site of minor trauma or injury to the skin [7–11]. The algae may spread from the intestinal tract or other primary sites of infection, to other organs, including the brain, eye, kidneys, liver, and heart producing a systemic disease with high mortality (up to 75% in disseminated cases) [7, 12–14]. Clinically, canine protothecosis presents with intermittent hematochezia, anorexia, and emaciation. Skin lesions can occur as the sole clinical sign of disease or be accompanied by gastrointestinal, neurological, or ophthalmic signs. Canine protothecosis has mainly been caused by *P. bovis* [11, 12, 15]. Infections due to *P. wickerhamii* seems to be more rare [13].

*Prototheca* species have been tested against a wide range of antimicrobial agents demonstrating a very diverse susceptibility in vitro [17–19]. Furthermore, the lack of correlation between in vitro and in vivo drug activities has become a hallmark of protothecosis. In dogs, despite using a plethora of antimicrobial agents, the disease often leads to a poor outcome, especially if developed as a disseminated infection, and if proper diagnosis and treatment have been delayed [7, 15].

There are few reports of protothecosis in dogs. The largest study on canine protothecosis was carried out in Australia. Of the 17 reported cases, only two were *P. wickerhamii* [15]. In a recent study of 11 documented cases of protothecosis, three dogs had a molecular diagnosis of *P. wickerhamii* causing skin lesions, one in Australia and two in Italy [16]. In Brazil, only two well-documented reports of canine protothecosis have been published, both caused by *P. bovis* [11, 21].

This report describes an uncommon case of cutaneous infection caused by *P. wickerhamii* in a dog, underlining the refractoriness of the algae to conventional therapy and stressing the relevance of the condition to human health, owing to the zoonotic potential of the pathogen.

## Case presentation

An 18-month-old, intact female dog of mixed breed, was admitted to the veterinary clinic of the Pontifícia Universidade Católica do Paraná (PUC-PR), Curitiba, Brazil, with a 2-month history of cutaneous lesions. No other clinical signs were reported. The owner told that the dog had access to an outside area, and contact with humid environment, including leaf mold, and effluent from a sewage disposal plant.

**Fig. 1:**
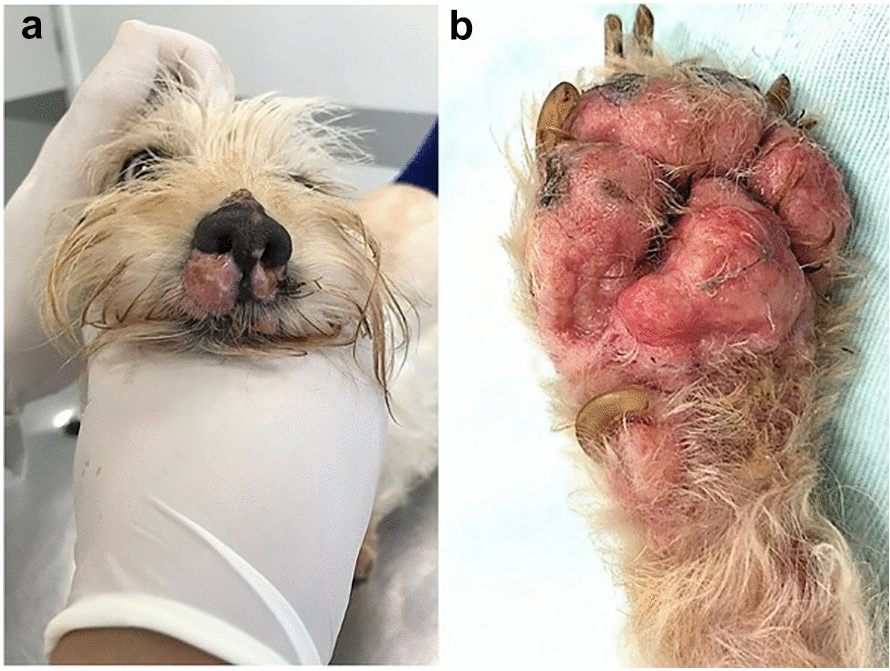
Cutaneous lesions caused by *Prototheca wickerhamii* in a dog. Dog, female, 18 months old with cutaneous protothecosis: **a** eroded-ulcerative and exudative nasolabial plaques; **b** erythematous, ulcerative and painful lesions on foot pad

Upon clinical examination, eroded, ulcerated, exudative, and painful lesions on nasal planum and foot pads (Fig. 1), associated with sneezing and bilateral sanguinopurulent nasal discharge were observed, as well as mandibular and prescapular lymphadenomegaly, detected on palpation.

The dog was subjected to standard blood and urine analysis, abdominal ultrasound and chest radiograph and neurological and ophthalmic examinations. Material collected by fine-needle aspiration from the nasal lesion and prescapular lymph nodes were submitted for Gram and Diff-Quick cytology staining. Biopsy fragments of foot pads and nasal planum regions were collected aseptically, and subjected to histopathological examination and microbiological culture. Tissue homogenates and urine were inoculated onto sheep blood agar medium (5%) and MacConkey agar medium (Oxoid™, Sao Paulo, Brazil), and incubated under aerobic conditions for 3 days at 37 ºC. Tissue specimens were also plated on Sabouraud agar medium (Oxoid™, Sao Paulo, Brazil) and incubated aerobically for 15 days at 37 °C.

The yeast-like colonies grown in culture were initially identified as *Prototheca*, based on conventional, phenotype-based methods (macro- and micromorphology and biochemical, carbohydrate assimilation tests) [22]. Confirmatory identification was performed by using matrix-assisted laser desorption/ionization time-of-flight mass spectrometry (MALDI-TOF MS, Bruker and DaltonicsTM, Bremen, Germany) at 337 nm laser wavelength. Spectra were analysed between 2000 and 20,000 m/z using FlexControl 3.3 software. Genus- and species-level identification was evaluated at threshold scores of  ≥ 1.7 and  ≥ 2.0, respectively [20]. Finally, the isolated strain was subjected to molecular speciation with a newly proposed PCR-based method targeting the mitochondrially-encoded cytochrome b (CYTB) gene marker [3].

Results of the haematological evaluation, serum biochemistry, and urine analyses were within normal range.

**Fig. 2:**
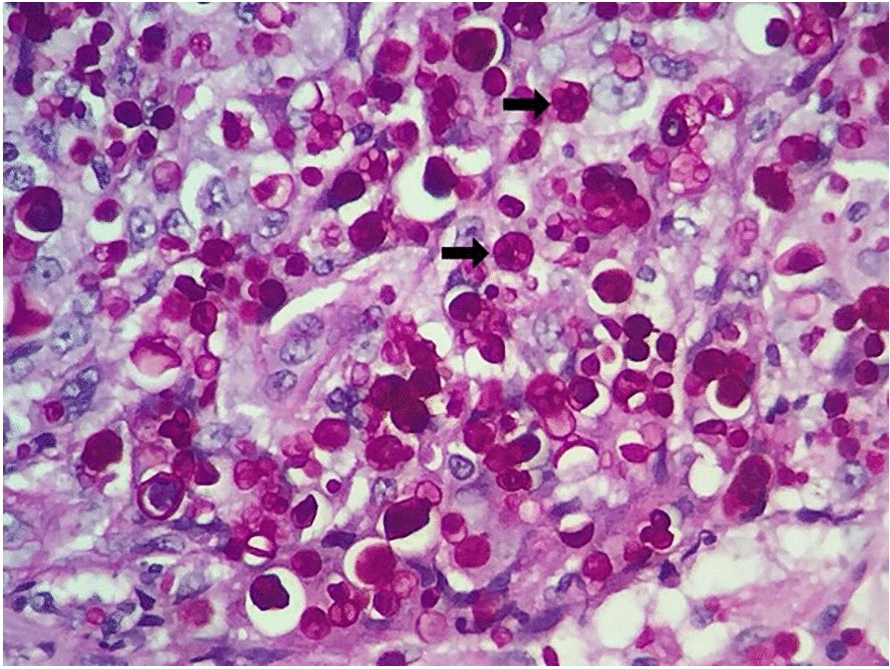
Histopathological presentation of cutaneous protothecosis. Dermatohistopathological examination of a dog, with cutaneous protothecosis, showing numerous encapsulated structures, rounded to oval (arrows), in the middle of the inflammatory infiltrate (PAS 100X)

Cytopathological examination (400 and 1000 × magnification) of prescapular lymph nodes and impression smear of the nasal lesion, revealed a pyogranulomatous infiltrate, although no infectious agent was observed. Upon histopathological examination at 1000 × magnification, oedema and intense inflammatory reaction with a diffuse distribution throughout the dermis were noted, with abundance of neutrophils, macrophages, lymphocytes, and plasma cells, containing numerous Periodic Acid Schiff -positive, encapsulated, spherical or oval structures, with a cartwheel-like appearance, ranging from 3.5 to 20 µm, thus morphologically compatible with *Prototheca* algae (Fig. 2).

Post 48 h incubation, on either Sabouraud or blood agar, greyish-white and opaque colonies, attaining a diameter of 1 mm were seen. Microscopically, single cells and endosporulating cells (moruloid sporangia aspect) were clearly visible, suggestive of *Prototheca wickerhamii.* The species identity was confirmed by both MALDI-TOF MS profiling and CYTB-based genotyping (PCR-sequencing).

**Fig. 3:**
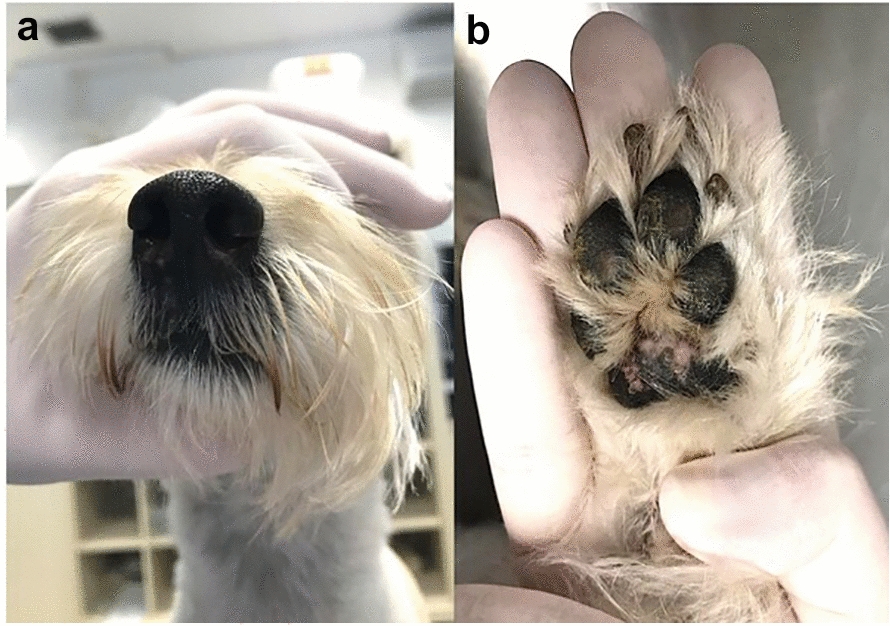
Cutaneous protothecosis after treatment. Involution of nasolabial plaques **a** and foot pad lesions **b** after initial treatment with itraconazole 10 mg/kg orally once daily after 60 days

Once the diagnosis was made, treatment with oral itraconazole was started at a dose of 10 mg/kg, once daily. Although itraconazole alone presents a poor response, the literature presents it as a possible option as a single agent in the treatment of protothecosis in some cases [7]. The response was slow yet progressive and, after 60 days of treatment, lesion regression was observed (Fig. 3). Treatment was continued for six months with clinical and blood follow-ups.

**Fig. 4:**
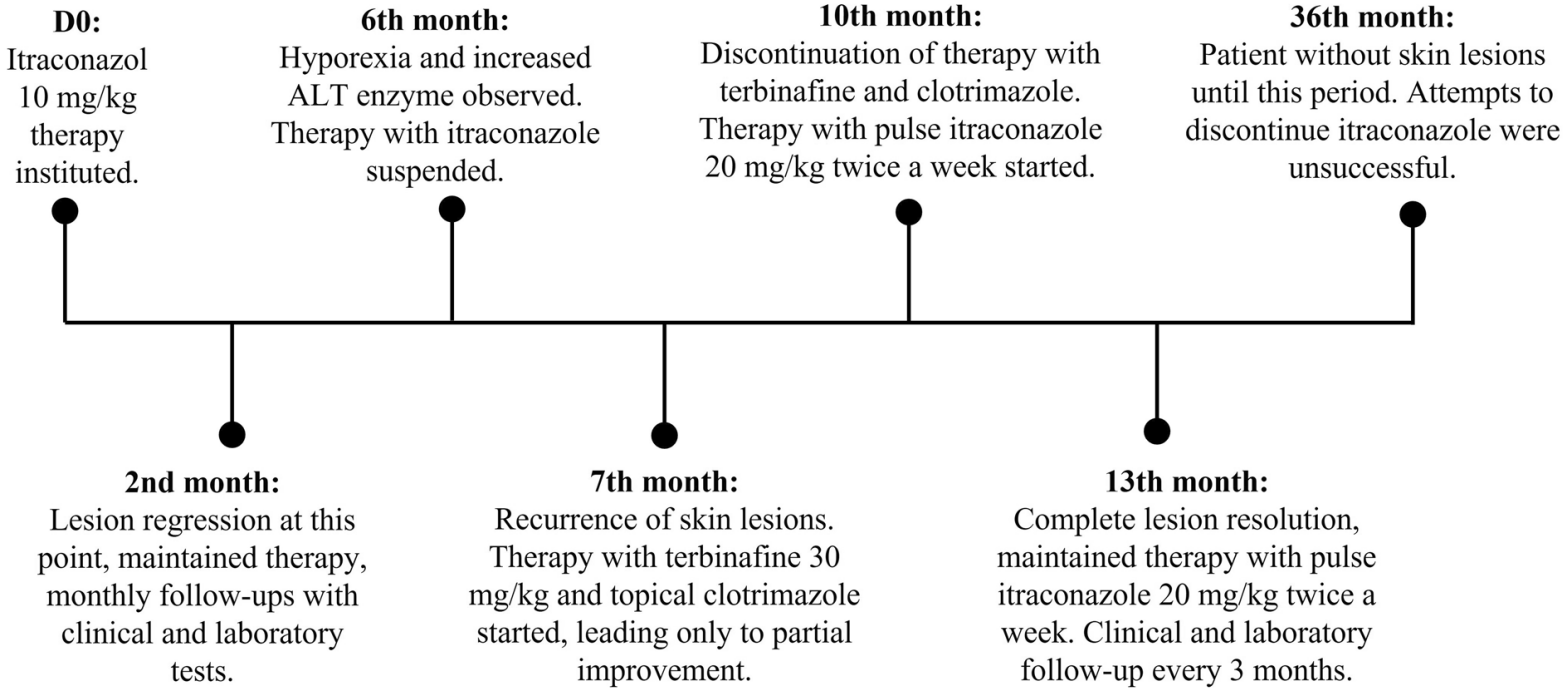
Treatment timeline. Treatment instituted according to elapsed time. D0 (day zero) = diagnosis and start of treatment and other times presented in months

Six months after starting treatment with itraconazole, the dog presented hyporexia and an increase in hepatic alanine aminotransferase enzymes (from 65 U/L to 189 U/L, laboratory reference indicates normality up to 88 U/L). Due to the apparent improvement in the condition and the observed side effects, itraconazole (human generic) was then discontinued. Values normalized after 30 days of therapy with S-adenosyl-methionine (at a dose of 20 mg/kg once a day orally. After 30 days of discontinuation of itraconazole), however, the clinical signs in the nasal planum and foot pad recurred. Consequently, treatment with oral terbinafine (human generic) at a dosage of 30 mg/kg, once daily, and topical clotrimazole (Canesten^®^, Bayer, 10 mg/g) on the foot pad lesions were instituted. The dog remained on treatment with terbinafine for three months, with partial regression of the lesions, which prompted the interruption of the drug. Thence, oral itraconazole was again prescribed in a pulse regimen (i.e., intermittent administration of a drug at the recommended dose with a longer-than-accepted interval between doses) at a dose of 20 mg/kg/day for two consecutive days a week. This pulse regimen led to clinical improvement, with complete resolution of cutaneous lesions after 3 months and no side effects were observed. The dog remains under 3-month follow-up monitoring with clinical examination, blood count, serum biochemistry and remains without skin lesions. Attempts to discontinue itraconazole in pulse twice a week were not successful with relapse within a few days of disuse. Figure 4 shows the treatment timeline.

## Discussion and conclusions

The common failure of treatment of canine protothecosis relates to several factors, with the immunological impairment of the host, delayed diagnosis, and drug resistance of the pathogen being the most widely recognized [11].

A number of antibacterial, antifungal, and algaecidal compounds have been evaluated in vitro [17–19] and in vivo [7, 15] with the aim of treating protothecosis in domestic animals. However, none of the compounds tested so far, have produced an efficient and consistent clinical response. In the present report, the initial treatment with oral itraconazole resulted in regression, albeit very slow, of the lesions. However, even the drug was maintained for 6 months since the lesions cleared, they reappeared shortly after discontinuation of therapy. The complete clinical cure, with no recurrence over 30 months follow-up, was obtained only after a long-term (3 months) treatment with itraconazole at twice higher dose (20 mg/kg) in pulse intermittently. It thus seems that itraconazole exerts algaestatic rather than algaecidal effect on *Prototheca* cells, when applied in suboptimal dosage. The increased dosage of itraconazole might have promoted therapeutic concentration, augmenting its algaecidal effect on the algae in the target tissue.

The high cutaneous concentrations achieved by itraconazole after oral administration and its pharmacokinetic profile suggest pulse administration. This has been proven successful in the treatment of *Malassezia pachydermatis* and dermatophytosis skin infections in dogs [23, 24]. The present case is the first that used pulsed itraconazole for the treatment of cutaneous protothecosis in dogs.

The benefits of pulse administration, when compared to daily administration, include a lower potential for side effects and adverse reactions, increased owner compliance, and reduced cost of treatment [23].

The present report describes the failure of terbinafine to cure the protothecal infection. Administration of terbinafine has been advocated as a treatment for human protothecosis of the skin, when patients do not respond to azole therapies [5]. It should be noted that in the only report that used terbinafine for the treatment of canine protothecosis, despite its in vitro activity, the drug partially controlled the lesions but failed to contain the infection, leading to the animal’s death [13].

The prognosis of cutaneous protothecosis in dogs is much better than that of systemic disease. Also, *P. wickerhamii* is generally more susceptible to treatment than *P. bovis* [2, 15]. Still, as demonstrated in our case, *P. wickerhamii* may resist conventional treatment regimens and persist in animal tissues for weeks to months. This is the first time that cutaneous lesions in dogs inflicted by *P. wickerhamii* have been cured successfully by using pulse regimen itraconazole treatment.

Protothecosis in both dogs and humans has often been associated with immunosuppressive conditions [7]. In the present case, blood and urine analyses, abdominal ultrasound and chest radiograph showed no changes, reinforcing the evidence that neither local nor systemic immunosuppression was present and indicating that protothecal cutaneous lesions may develop in healthy animals. This is in line with previous findings that dogs with cutaneous infection, as the sole clinical manifestation, did not display any immunological defects whatsoever [8, 10].

Whereas, certain inadequacies in farm management, such as poor hygiene of housing, feeding, and milking have long been considered as predisposing factors to protothecosis in dairy cattle [11], those favoring the disease development in dogs and cats, are yet to be disclosed.

The main route of protothecal infections in dogs is probably by ingestion of contaminated water or food [11, 12, 15]. Alternatively, the pathogen may infect the animal through skin or mucosal abrasions or other minor trauma [1, 2]. In our report, given the presence of nasolabial plane and foot pad lesions, it is possible that the dog may have developed the infection through a direct contact with *Prototheca*-infested outdoor environment, especially that it included sources conducive for the protothecal growth (e.g., untreated sewage effluent).

Cutaneous protothecosis, with no clinical signs originating from other organs is uncommon in dogs [9]. Our case was such a case with clinical signs restricted exclusively to the nasal plane and foot pads of the animal. Macro-and microscopic aspects of the skin lesions were similar to those observed in other dogs with cutaneous involvement [8, 13].

A wide spectrum of molecular methods that have become available, over the last decade, provide a powerful complement to conventional measures used for fast and reliable identification of *Prototheca* species. Currently, the only diagnostic tool, used also in this study, allowing for an accurate differentiation between all *Prototheca* species is PCR-sequencing of the partial CYTB gene [3].

This report highlights the refractoriness of skin infections by* Prototheca wickerhamii* with therapies proposed in the literature and suggests a new treatment option with oral itraconazole in pulse dosing for long-term disease control successfully performed in a dog with skin lesions.

## Data Availability

The datasets used and analyzed in the current study are available from the corresponding author on reasonable request.

## Abbreviations

CYTB: Cytochrome B
MALDI-TOF: Matrix-assisted laser desorption-ionisation-time of flight mass
MS: Spectrometry
PAS: Periodic acid schiff
