# Correction to: Cutaneous protothecosis in a dog successfully treated with oral itraconazole in pulse dosing

**DOI:** 10.1186/s13028-023-00682-1

**Published:** 2023-06-22

**Authors:** Vanessa Cunningham Gmyterco, Tomasz Jagielski, Gustavo Baldasso, Louise Helene Bacher, Marcio Garcia Ribeiro, Marconi Rodrigues de Farias

**Affiliations:** 1 Department of Veterinary Medicine, School of Life and Sciences, Pontificia Universidade Catolica do Parana, 1155 Imaculada Conceicao Street, Curitiba, PR 80215-901 Brazil; 2 Department of Medical Microbiology, Institute of Microbiology, Faculty of Biology, University of Warsaw, I. Miecznikowa 1, 02-926 Warsaw, Poland; 3 Department of Animal Production and Preventive Veterinary Medicine, School of Veterinary Medicine and Animal Sciences, Sao Paulo State University-UNESP, Botucatu, SP 18618-681 Brazil

**Correction to:** ***Acta Vet Scand*** **65, 7 (2023)**

10.1186/s13028-022-00662-x

Following publication of the original article [1], the authors reported an error in postal code of Affiliation 2. The correct postal code is 02-926.

Moreover, the authors detected a typo in the caption of Fig. 1. The updated caption is given below and the changes have been highlighted in **bold typeface**.

Figure 1 Cutaneous lesions caused by *Prototheca****wickerhamii*** in a dog. Dog, female, 18 months old with cutaneous protothecosis: a eroded-ulcerative and exudative nasolabial plaques; b erythematous, ulcerative and painful lesions on foot pad.

The original article [1] has been updated.
